# Tumeur fibreuse solitaire de la prostate: à propos d’un cas

**DOI:** 10.11604/pamj.2021.39.285.30406

**Published:** 2021-08-31

**Authors:** Dalila Ahnou, Abdelwahab Belkacem-Nacer, Mustapha Boubrit

**Affiliations:** 1 Service de Radiologie, Université Alger 1, Alger, Algérie,; 2 Service d´Urologie, Université Alger 1, Alger, Algérie

**Keywords:** Tumeur fibreuse solitaire, prostate, IRM, prostatectomie, à propos d’un cas, Solitary fibrous tumor, prostate, MRI, prostatectomy, case report

## Abstract

La tumeur fibreuse solitaire est une tumeur mésenchymateuse souvent bénigne et rare décrite pour la première fois dans la plèvre, la localisation prostatique est exceptionnelle. Nous rapportons le cas d´un patient de 77 ans qui a consulté pour symptômes du bas appareil urinaire à type de dysurie et pollakiurie. Le scanner et l´imagerie par résonance magnétique (IRM) ont montré l´origine prostatique de la lésion et précisé les rapports de cette masse avec les structures adjacentes saines, élément important pour la résécabilité de la tumeur. La biopsie transrectale avec une étude immunohistochimie ont confirmés le diagnostic, les cellules tumorales exprimaient le CD34, Bcl2 et CD 99. Le traitement était chirurgical par prostatectomie radicale.

## Abstract (en)

Solitary fibrous tumour is usually a benign and rare mesenchymal tumour. The first case was reported in patients with pleural involvement. Solitary fibrous tumour of the prostate is exceptional. We here report the case of a 77-year-old patient presenting with lower urinary tract symptoms such as dysuria and polalkiuria. CT scan and MRI showed the prostate lesion and determined its relation to adjacent healthy structures, an important factor in evaluating tumour resectability. Transrectal biopsy with immunohistochemical examination confirmed the diagnosis; CD34, Bcl2 and CD 99 were expressed. Radical prostatectomy was performed.

## Introduction

La tumeur fibreuse solitaire (TFS) est une tumeur rare décrite pour la première fois dans la plèvre [1]. Les localisations urogénitales sont exceptionnelles, le diagnostic est principalement fait par immunohistochimie. Nous rapportons ici une observation de localisation prostatique.

## Patient et observation

**Informations relatives au patient:** M. Aissa âgé de 77 ans a consulté pour une pollakiurie avec dysurie sans hématurie évoluant depuis deux mois

**Résultats cliniques:** le toucher rectal, retrouvait une masse dure prostatique gauche. Le bilan biologique était normal, l´examen cytobactériologique des urines (ECBU) ne retrouvait pas d´infection urinaire et le taux sérique de l´antigène spécifique prostatique était de 6.3 ng/ml.

**Figure 1:**
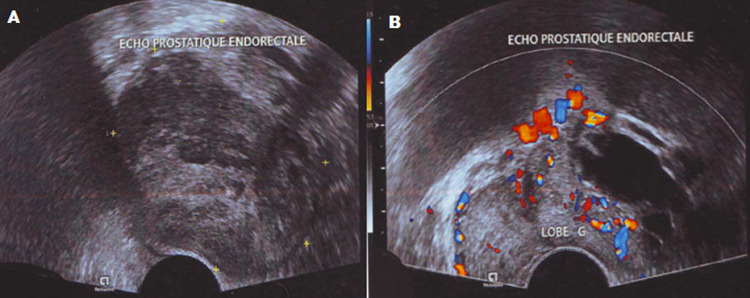
échographie endorectale en mode B (a) et Doppler couleur; (b) formation tumorale hétérogène vascularisée au Doppler couleur du lobe gauche de la prostatique

**Figure 2:**
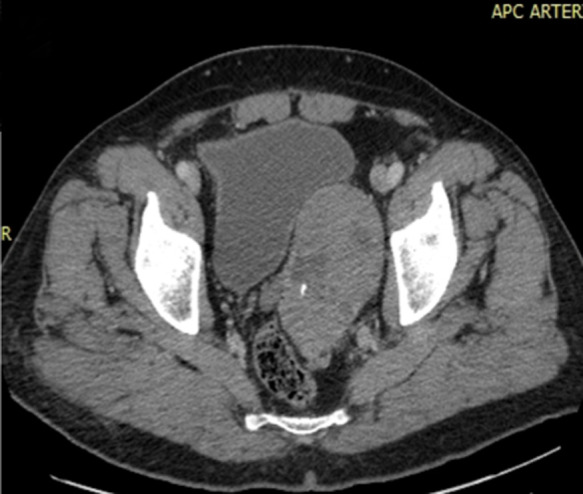
homme de 77ans; coupe scanner axiale; masse pelvienne postéro-latérale gauche bien limitée ovalaire prostatique probable refoulant la vessie en avant et la face antéro-latérale gauche du rectum, se rehausse de manière hétérogène, contenant des zones hypo denses et des calcifications

**Démarche diagnostique:** l´échographie par voie sus pubienne et endorectale a mis en évidence une formation tumorale, hétérogène, solide et kystique vascularisée au Doppler couleur refoulant la face postéro-latérale gauche de la vessie, et la face antérieure du rectum sans signe d´infiltration. La composante kystique envahit le lobe gauche de la prostate, la vésicule séminale homolatérale est écrasée et laminée (Figure 1). Le scanner abdomino-pelvien avec injection de produit de contraste iodé montrait une volumineuse masse pelvienne gauche, ovalaire bien limitée se rehaussant de manière hétérogène après contraste, ménageant des petites zones de nécrose et contenant des calcifications, elle arrivait au contact du rectum en arrière, de la vessie en avant avec conservation d´un liseré graisseux de séparation entre eux, faisant évoquer une origine prostatique ou de la vésicule séminale gauche, car cette dernière n´était pas identifiable (Figure 2).

**Figure 3:**
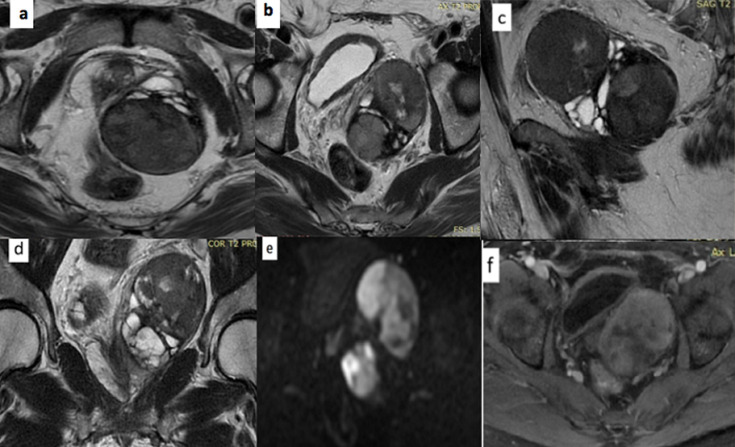
IRM séquences pondérées en T2 axiales (a,b) sagittale (c) et coronale (d) montrait une masse ovalaire bien limitée, polylobée prostatique gauche solido-kystique en hypo signal T1, en signal T2 intermédiaire ménageant des zones kystiques en (d), en hyper signal diffusion et en (e) se rehaussant de façon hétérogène après contraste(f); absence d´envahissement vésicale ou rectale ou de la vésicule séminale droite, absence d´envahissement ganglionnaires

Il n´y avait pas d´urétéro-hydronéphrose et il n´existait pas de localisation ganglionnaire, pulmonaire, hépatique ou osseuse. L´IRM réalisée pour mieux caractériser cette lésion, a mis en évidence une masse pelvienne latéralisée à gauche d´origine prostatique solido kystique de signal hétérogène en hyposignal T1, en signal intermédiaire en T2, en hyper signal diffusion rehaussée après injection de gadolinium. De forme ovalaire bien limitée mesurant 108x82x59 mm de grands axes. La vésicule séminale gauche n´est pas individualisée (Figure 3). Le lobe droit de la prostate et la vésicule séminale homolatérale étaient sans anomalies. Cette masse refoule la vessie à droite, le rectum en arrière, sans signe d´envahissement. Par ailleurs on ne retrouvait pas d´adénopathies pelviennes péjoratives.

**Figure 4:**
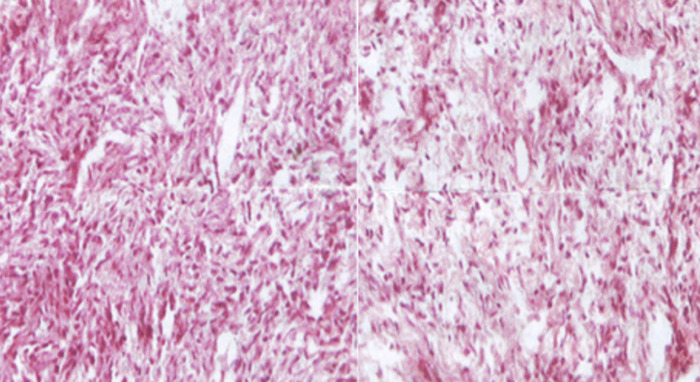
coloration à l´hématoxyline éosine montre une prolifération mésenchymateuse constituée de cellules fibroblastiques aux cytoplasmes mal définis et aux noyaux fusiformes onduleux en “en vagues” les mitoses sont peu nombreuses (<1 mitoses/10 champs au G x100) le stroma conjonctif lâche inclut de nombreuses veinules et capillaires sanguins, aux parois myélinisées

Le diagnostic de tumeur fibreuse solitaire a été posé après biopsie transrectale. L´immunohistochimie a montré que les cellules exprimaient fortement CD34 et anti-Bcl2 et étaient colorées positives pour l´expression de CD 99, mais négatives pour les récepteurs: de la progestérone (PR), de l´actine des muscles lisses (AML), de l´anti-pancytokeratine (AE1/AE3), du PS100 et anti-CD117 éliminant ainsi une tumeur stromale primitive de la prostate, un processus carcinomateux, une tumeur nerveuse de la gaine périphérique et une tumeur stromale gastro-intestinale (Figure 4).

**Intervention thérapeutique et suivi thérapeutiques:** le patient a été opéré, il a bénéficié d´une prostatectomie radicale avec curage ganglionnaire ilio-obturateur bilatéral. La pièce de prostatectomie radicale était déformée d´aspect bilobé, le lobe gauche était déformé par une néoformation tumorale bien limitée d´aspect plein blanchâtre à la coupe. Les vésicules séminales, les canaux déférents et le lobe droit de la prostate étaient de taille et de morphologie normale. L´étude microscopique retrouvait un parenchyme prostatique siège d´une prolifération mésenchymateuse bien circonscrite par une capsule conjonctive avec des marges chirurgicales saines avec un profil immunohistochimie d´une tumeur fibreuse solitaire (CD34, Bcl2, CD99 positifs).

## Discussion

Lee *et al*. et Chick *et al*. ont été les premiers à décrire les tumeurs fibreuses solitaires (TFS) au niveau de la plèvre avec une incidence de 2.8 pour 100000 et représentent moins de 2% des tumeurs des tissus mous [1, 2]. Ce sont des tumeurs mésenchymateuses ubiquitaires développées à partir des fibroblastes présents dans le tissu conjonctif sous mésothelial (tumeur myofibroblastiques) décrites dans des sites varies: le rein, le foie, le pancréas le médiastin, l´orbite, la glande thyroïde et la cavité nasale, elles sont bénignes ou de faible malignité et pouvant récidiver [3, 4]. Il a été rapporté que 16% des TFS sont de localisations pelviennes, l´atteinte prostatique est très rare avec moins de 30 cas rapportés dans la littérature chez des patients dont l´âge varie entre 21 et 75 ans [5, 6].

La tumeur peut être, asymptomatique, mais lorsqu´elle est volumineuse comme dans notre cas, elle est à l´origine de rétention urinaire, de dysurie ou de constipation. Le dosage des marqueurs prostatiques (PSA) est le plus souvent normal. La taille de ces tumeurs varie de 2 à 14 cm, elles sont expansives sans signes d´infiltrations [7, 8], dans notre cas la tumeur mesurait plus de 10 cm de diamètre et le bilan locorégional était négatif. L´aspect radiologique des TFS n´est pas spécifique, le scanner et l´IRM permettent de faire le bilan d´extension local et général. L´échographie révèle une lésion généralement hypo échogène, mais pouvant être hétérogène par la présence de zones de dégénérescence myxoïde souvent hyper vasculaire, c´est le cas dans notre observation avec une hyperhémie au Doppler couleur. Elle apparait à l´examen tomodensitométrique comme une masse lobulée dont l´hétérogénéité varie selon la présence de remaniements nécrotiques, kystiques ou calcique, le rehaussement est variable après injection d´iode.

L´IRM affirme le site d´origine et les rapports de cette masse avec les structures adjacentes saines, élément important pour la résécabilité de la tumeur. La plupart des TFS prostatiques sont iso intenses en T1 et variables en T2, mais les plus volumineuses peuvent être hyper intenses et hétérogènes sur les séquences pondérées en T2. Cette hétérogénéité est retrouvée dans notre observation, l´hypointensité en T2 serait attribuable à la forte teneur en fibres collagènes qui présenteraient un rehaussement retardé alors que les zones hyper intenses sont hyper cellulaires et prendraient fortement le contraste [9, 10]. Dans la plupart des cas, le diagnostic est confirmé par une étude histologique réalisée souvent à la suite d´une biopsie transrectale écho guidée.

Le diagnostic est anatomopathologique, l´étude immuno histochimique apporte d´importants éléments d´orientation, la négativité des marqueurs à l´actine du muscle lisse (AML) ou à la désmine permet de les distinguer des tumeurs musculaires. L´absence d´immuno-marquage des cellules tumorales vis-à-vis de l´anticorps anti-pancytokeratine (AE1/AE3) élimine un éventuel processus carcinomateux type “carcinome sarcomatoïde”, la négativité vis-à-vis des anticorps anti-CD117, pour la progestérone et pour l´anticorps anti-PS100 permettent d´écarter une tumeur gastro-intestinale (GIST), une tumeur stromale primitive, une tumeur maligne des gaines nerveuses ou un mélanome. Mais c´est le CD34 qui reste le marqueur tumoral le plus fiable retrouvé constamment dans les tumeurs fibreuses solitaires comme c´est le cas dans notre observation. Le traitement de cette tumeur est chirurgical, la prostatectomie est utilisée comme traitement radical, le pronostic est souvent favorable, une surveillance est indiquée, car le risque récidive est possible.

**Perspectives du patient:** le patient a été revu à trois et six mois, avec bonne suite opératoire, aucune récidive n´est constatée jusqu´à maintenant.

**Consentement éclairé:** le patient a donné son consentement éclairé.

## Conclusion

Les tumeurs fibreuses solitaires sont le plus souvent situées dans la plèvre. La localisation prostatique est rare, l´IRM est beaucoup plus sensible que la TDM et l´échographie pour délimiter l´origine de la tumeur, la présence de foyers hypointenses en T1 et en T2 est très évocatrice. L´examen anatomopathologique, complété par une étude immunohistochimie permettent de confirmer le diagnostic.
